# Neurofibroma invading into urinary bladder presenting with symptoms of obstructed defecation and a large perineal hernia

**DOI:** 10.1186/1471-2482-14-21

**Published:** 2014-04-16

**Authors:** Duminda Subasinghe, Chathuranga Tisara Keppetiyagama, Chandu De Silva, Neville D Perera, Dharmabandhu N Samarasekera

**Affiliations:** 1 The National Hospital of Sri Lanka, University Surgical Unit, Colombo, Sri Lanka; 2 Department of Pathology, University of Colombo, Colombo, Sri Lanka; 3 Department of Urology and renal transplant, The National Hospital of Sri Lanka, Colombo, Sri Lanka

**Keywords:** Multiple neurofibromatosis, Obstructive defecation, Perineal hernia

## Abstract

**Background:**

Pelvic floor hernias pose a diagnostic and a treatment challange. Neurofibromatosis is a rare systemic disease, and urinary tract involvement is rare.

**Case presentation:**

Here we report a case of a 54-year-old female with multiple neurofibromatosis who presented with features of obstructed defecation and was found to have a large perineal hernia. At surgery, we found an unusual herniation of a large neuropathic bladder and rectum through a perineal defect. She underwent reduction cystoplasty and repair of the pelvic floor using a prolene mesh. Subsequent histopathological examination confirmed a large neurofibroma infiltrating the urinary bladder.

**Conclusion:**

Neurofibromatosis of the bladder is rare it should be considered as a differential diagnosis in patients presenting with symptoms of obstructed defecation.

## Background

Perineal hernias are rare and to date, about 100 cases have been reported in the literature [1]. It represents the protrusion of intra peritoneal or extra peritoneal contents through a congenital or an acquired defect in the pelvic floor. The first case of an acquired perineal hernia was reported in 1939 by Yeomen [2] and that of a primary hernia was reported by De Garangeot in 1743 [3]. This rare condition could be a diagnostic and a management challenge. These conditions need to be differentiated from other causes of perineal pathologies such as lipoma, bartholian cyst etc. Acquired perineal hernias are primary or secondary. Primarily acquired perineal hernias are caused by factors associated with increased intra-abdominal pressure. They are more common in females as a result of the broader pelvis and the strectching of the pelvic floor during pregnancy and childbirth. Secondarily, acquired perineal hernias are incisional hernias associated with extensive pelvic operations such as abdominoperineal resection, pelvic exenteration and perineal prostatectomy. Here we describe a patient with type 1 multiple neurofibromatosis who presented with features of obstructed defecation and a perineal hernia. She underwent surgery and was found to have the urinary bladder infiltrated with neurofibroma on subsequent histopathology.

## Case presentation

**Figure 1:**
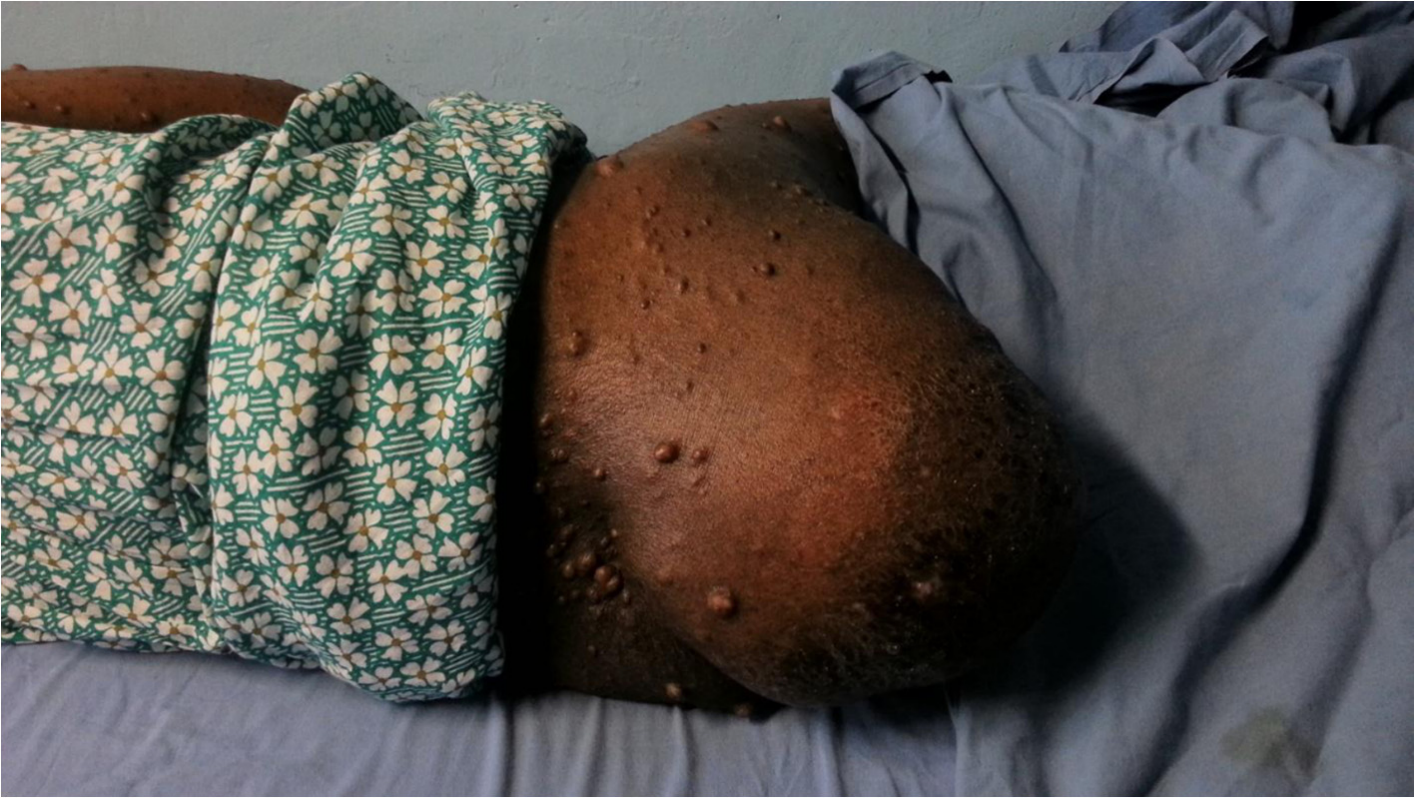
Large R/sided buttock hernia.

A 54 year old female presented with a history of a swelling in her right buttock of 6 years duration. Her main complaint was difficulty in defecation when sitting due to a large lump in the perineum (Figure 1). She also had features of obstructed defecation syndrome such as excessive straining with unsuccessful attempts to evacuate, prolonged episodes in the toilet, rectal pain, rectal digitation and laxative dependency. Obstructed defecation symptoms were not relieved by regular use of laxatives. She had no obstructive urinary symptoms. She had not undergone any major gynecological procedures.

She was a diagnosed patient with multiple neurofibromatosis type 1 since the age of 17 years and had undergone excision of neurofibromas adjacent to right knee joint and over the right buttock 7 years back. She did not have a family history of neurofibromatosis. She did not give a past history of hypertension or other comorbidities. On examination, she had multiple neurofibromas all over the body and one plexiform neurofibroma over left knee area. There was a non reducible lump of 25× 20 cm size over the right buttock. It had an expansile cough impulse and was non tender on palpation. Digital rectal examination was normal. Therefore, a clinical diagnosis of a secondary perineal hernia was made.

**Figure 2:**
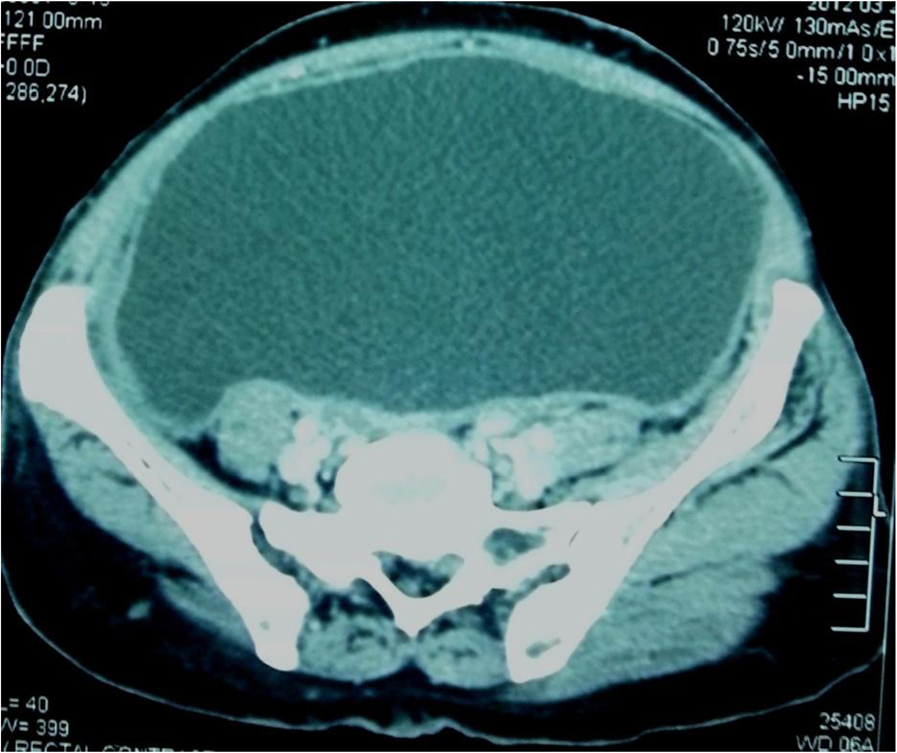
CT pelvis showing large neuropathic urinary bladder.

**Figure 3:**
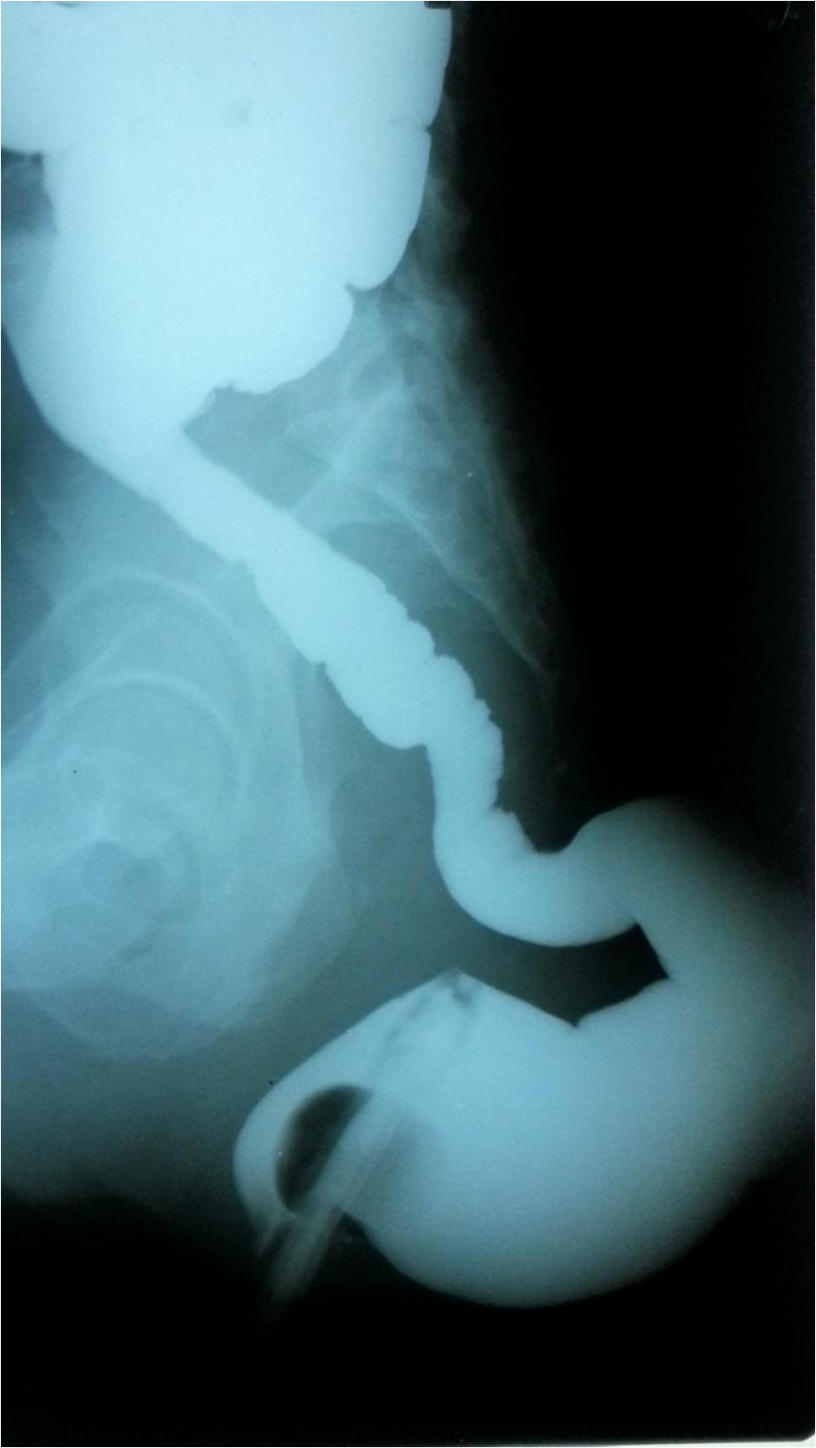
Barium enema showing the herniated rectum.

Contrast enhanced computed tomography showed a large poorly enhancing mass in the region of right ischiorectal fossa suggestive of herniation of a large neuropathic bladder (Figure 2) and rectum through right ischiorectal fossa. Her US scan of the abdomen revealed mild right sided hydronephrosis. She underwent sigmoidoscopy which was normal up to the proximal sigmoid colon. Barium enema (Figure 3) showed herniation of the rectum through a defect in the pelvic floor.

**Figure 4:**
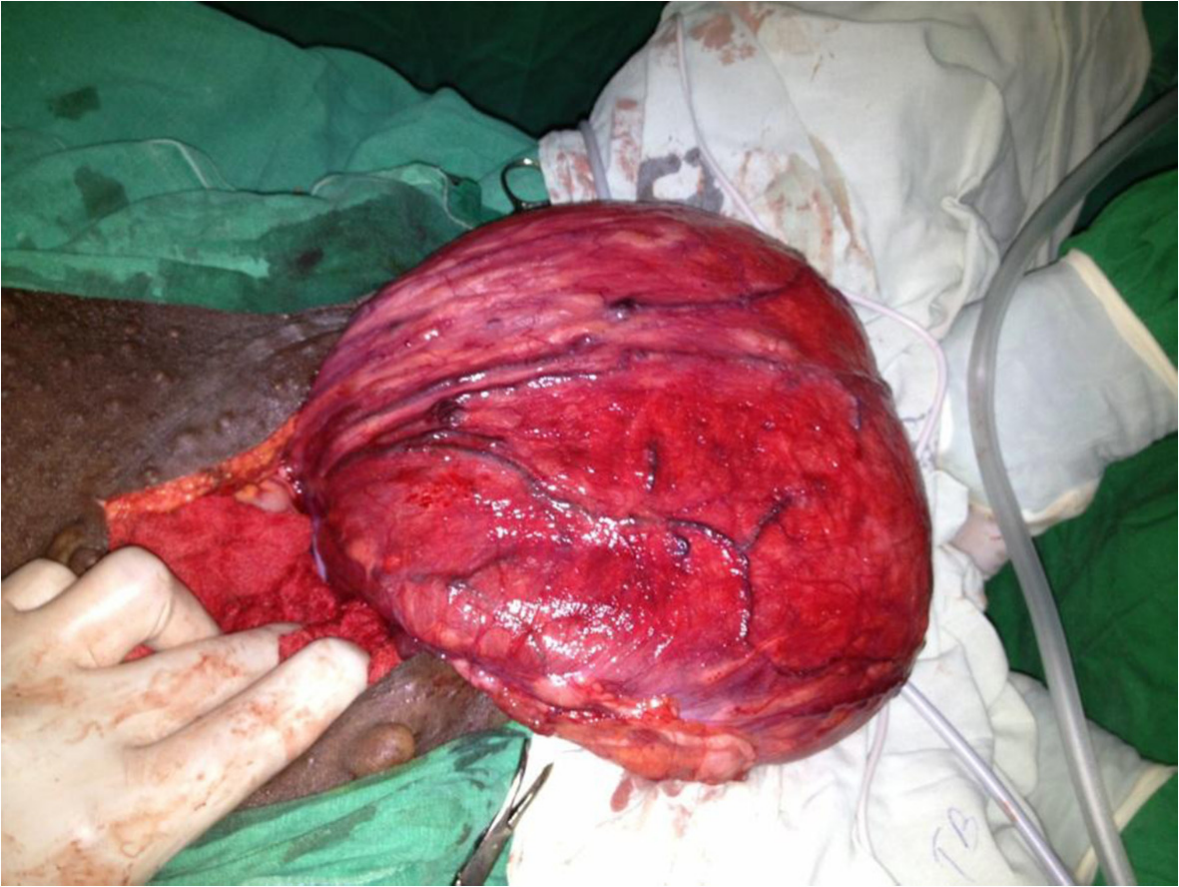
Large neuropathic bladder found during surgery.

**Figure 5:**
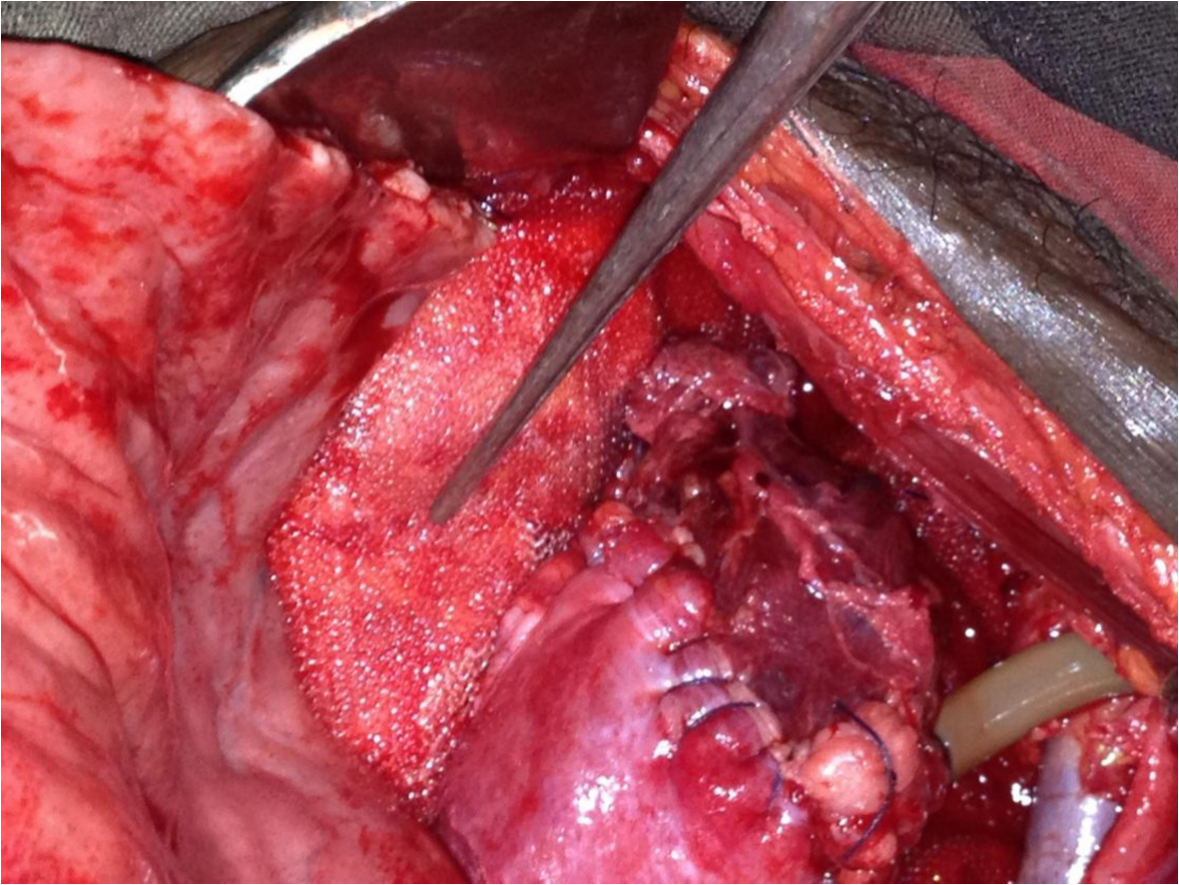
Repaired bladder with supra pubic catheter and mesh placed in the pelvic floor defect.

The patient was planned for a hernia repair by a combined surgery with participation of colorectal, urological and plastic surgical teams. The patient was positioned on Lloyd Davies position and the repair of her hernia was done using an abdominal approach with a lower mid line incision. There was a large hernia sac extending in to right buttock area which contained a large neuropathic urinary bladder (Figure 4). A defect was noticed in the levator ani posteriorly on the right side, through which the urinary bladder and a portion of rectum had herniated. Openings of the ureters were not demonstrated and the right ureter was traced up to pelvic brim and stenting done. Cystectomy and reduction cystoplasty was followed by suprapubic catheterization (Figure 5). Hernial orifice was repaired with a prolene mesh. She was discharged six weeks following surgery. Currently she is on regular clinic follow up and intermittent urethral catheterization training.

## Discussion

NF-1 is an autosomal dominant disorder characterized by multiple neurofibromas, multiple cutaneous café au lait spots, axillary freckling, optic nerve gliomas and skeletal abnormalities. It is one of the more common genetic disorders with a frequency of 1 in 3000 live births [4-6]. It is a multisystem disorder that may affect any organ in the body [7]. Visceral involvement in disseminated NF is rare. Involvement of the urinary tract by neurofibromatosis is uncommon. Neurofibromas of the genito-urinary tract have been shown to derive from nerves of the pelvic, vesicle and prostatic plexuses. The bladder is the commonest affected organ in the urinary tract, either as an isolated mass or a diffuse infiltrative process as in the cases described in this report. In 1878 Gerhardt [8] first described urinary tract involvement with a case of neurofibromatosis in the bladder and spinal cord and more than a century later a total of 75 cases have been reported of which less than a third are paediatric [9]. So far only four cases of malignant transformation of neurofibromatosis of the bladder have been reported [10-12]. Urinary bladder neurofibroma may present with LUTS, flank pain or enuresis and incontinence. But our patient presented with a large buttock mass and features of obstructed defecation syndrome.

Imaging plays an important role in the diagnosis, evaluation and follow-up of patients with abdominal manifestations of NF1. Barium studies may demonstrate intraluminal mass lesions. Our patient’s barrium enema was suggestive of a displaced rectum and sigmoid colon with herniation of rectum through the pelvic floor. Anatomically, perineal hernias are classified as anterior or posterior, depending on the relationship to the transverse perineal muscle. The posterior is the less common variety and occurs through a defect in the levator ani muscle or between the levator ani and the coccygeus muscle [13,14]. Our patient had a posterior perineal hernia where there was a defect in the levator ani muscle.The techniques of repair through a transabdominal, a perineal, or combined transabdominal and perineal approaches have been described. Principles of surgical repair includes reduction of the sac contents and repair of the pelvic floor defect with either direct suture or implantation of a mesh [13].

## Conclusions

Eventhough neurofibromatosis of the bladder is rare it should be considered as a differential diagnosis in patients presenting with symptoms of obstructed defecation specially with evidence of von Recklinghausen’s disease. According to the authors’ knowledge, this is the first report of neurofibromatosis involving the urinary bladder presenting as a buttock hernia causing obstructed defecation.

## Consent

Written informed consent was obtained from the patient for publication of this case report and any accompanying images. A copy of the written consent is available for review by the Editor-in-Chief of this journal.

## Competing interests

The authors declare that they have no competing interests.

## Authors’ contribution

All authors contributed to management of the patient and contributed equally to drafting of the manuscript. DNS and DS provided overall supervision and edited the final version of the manuscript. All authors have read and approved the final manuscript.

## Pre-publication history

The pre-publication history for this paper can be accessed here:

http://www.biomedcentral.com/1471-2482/14/21/prepub
